# Polymerization Degrees, Molecular Weights and Protein-Binding Affinities of Condensed Tannin Fractions from a *Leucaena leucocephala* Hybrid

**DOI:** 10.3390/molecules19067990

**Published:** 2014-06-12

**Authors:** Mookiah Saminathan, Hui Yin Tan, Chin Chin Sieo, Norhani Abdullah, Clemente Michael Vui Ling Wong, Emilia Abdulmalek, Yin Wan Ho

**Affiliations:** 1 Institute of Bioscience, Universiti Putra Malaysia, 43400 UPM Serdang, Selangor, Malaysia; E-Mails: saminathan_82@yahoo.com (M.S.); sieo@upm.edu.my (C.C.S.); 2 Faculty of Applied Sciences and Computing, Tunku Abdul Rahman University College, 53300 Kuala Lumpur, Malaysia; E-Mail: hytan@acd.tarc.edu.my; 3 Faculty of Biotechnology and Biomolecular Sciences, Universiti Putra Malaysia, 43400 UPM Serdang, Selangor, Malaysia; E-Mail: norhani@upm.edu.my; 4 Institute of Tropical Agriculture, Universiti Putra Malaysia, 43400 UPM Serdang, Selangor, Malaysia; 5 Biotechnology Research Institute, Universiti Malaysia Sabah, Jalan UMS, 88400 Kota Kinabalu, Sabah, Malaysia; E-Mail: michaelw@ums.edu.my; 6 Department of Chemistry, Faculty of Science, Universiti Putra Malaysia, 43400 UPM Serdang, Selangor, Malaysia; E-Mail: emilia@upm.edu.my

**Keywords:** condensed tannins, NMR, degree of polymerization, Q-TOF LC-MS, molecular weight, protein-binding affinity, *Leucaena leucocephala*

## Abstract

Condensed tannins (CTs) form insoluble complexes with proteins and are able to protect them from degradation, which could lead to rumen bypass proteins. Depending on their degrees of polymerization (DP) and molecular weights, CT fractions vary in their capability to bind proteins. In this study, purified condensed tannins (CTs) from a *Leucaena leucocephala* hybrid were fractionated into five different molecular weight fractions. The structures of the CT fractions were investigated using ^13^C-NMR. The DP of the CT fractions were determined using a modified vanillin assay and their molecular weights were determined using Q-TOF LC-MS. The protein-binding affinities of the respective CT fractions were determined using a protein precipitation assay. The DP of the five CT fractions (fractions F1–F5) measured by the vanillin assay in acetic acid ranged from 4.86 to 1.56. The ^13^C-NMR results showed that the CT fractions possessed monomer unit structural heterogeneity. The number-average molecular weights (*M*_n_) of the different fractions were 1265.8, 1028.6, 652.2, 562.2, and 469.6 for fractions F1, F2, F3, F4, and F5, respectively. The *b* values representing the CT quantities needed to bind half of the maximum precipitable bovine serum albumin increased with decreasing molecular weight—from fraction F1 to fraction F5 with values of 0.216, 0.295, 0.359, 0.425, and 0.460, respectively. This indicated that higher molecular weight fractions of CTs from *L. leucocephala* have higher protein-binding affinities than those with lower molecular weights.

## 1. Introduction

**Figure 1:**
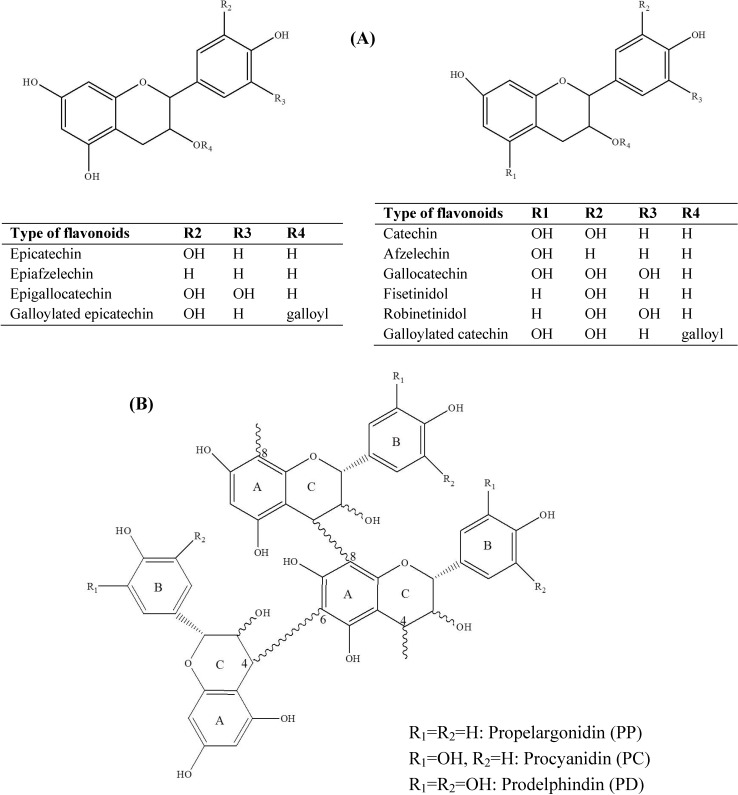
Chemical structures of a flavan-3-ol monomer (**A**) and condensed tannins (**B**).

Condensed tannins (CTs), also known as proanthocyanidins, are secondary plant metabolites. They are complexes of oligomers and high molecular weight polymers built up of flavan-3-ol monomer units (Figure 1), which are linked by C4–C6 or C4–C8 bonds that are not susceptible to cleavage by hydrolysis [1,2]. CTs are commonly found in tropical scrub legume plants from the genera *Leucaena*, *Acacia*, *Albizia*, *Prosopis*, *Desmanthus* and *Desmodium* [3]. They are also found in several important forage genera, such as *Lotus*, *Coronilla*, *Lespedeza*, *Hedysarum*, *Trifolium* and *Onobrychis* [4]. Significant CT diversity is apparent in the species of various genera. The diversity of CTs arises from the structural variability of the monomer units, extent of polymerization and stereochemistry. The average molecular masses of CTs range from 288 to >5000 Da [5]. The biological activities of plant CTs are recognized to be largely dependent on their molecular weight and structure [6,7].

In ruminants, CTs have long been recognized as “antinutritional” factors due to depression of feed intake, decrease in palatability, as well as reduction in dry matter (DM), fibre and nitrogen digestibility [8]. However, some studies have indicated that low levels of CTs may actually improve the utilization of feed proteins by ruminants and reduce the occurrence of bloat [9,10,11], and moderate concentrations (3%–4% DM) could improve milk production, ovulation rate, wool growth and reduce internal parasite burdens [12,13,14,15]. Recently, it was discovered that CTs of *Leucaena* have the potential to reduce methane gas production in ruminants [16,17]. CTs have also been found to exhibit protein‑binding ability and are able to protect proteins from being degraded in the rumen by forming CT-protein complexes. The CT-protein complexes are then hydrolyzed in the small intestine releasing the proteins for digestion and absorption [18]. It has been reported that the incorporation of CT extract from several species of *Leucaena* with the objective of binding proteins has increased the nitrogen digestibility of >78% in the small intestine of ruminants [19]. Generally, the bioactivity capacity of CTs to bind proteins depends on their molecular size [6]. Porter and Woodruffe [20] reported that the capacity of CTs to bind proteins is a function of the polymer chain length—the larger the CT, the greater the binding ability on a g/g basis. Osborne and McNeill [21] found that the larger-sized CTs of *L. pallida* and *L. trichandra* had stronger protein-binding capacity than the smaller-sized CTs.

*Leucaena leucocephala* (Lam.) de Wit is the most well known of all the *Leucaena* species. It is probably the most widely used tree legume in the World. It has a high protein content and could be used as a protein supplement for ruminants fed on low quality forages and crop residues. A study by Khamseekhiew [22] on two *L*. *leucocephala* hybrids (*Leucaena* hybrid Bahru and *Leucaena* hybrid Rendang), which were used as feed supplement for sheep in Malaysia, showed that their CTs have strong binding affinity for proteins. Later, Huang *et al.* [16] studied the protein-binding affinities of CTs of *Leucaena* hybrid Bahru and a local Malaysian *L. leucocephala* and found that the protein‑binding affinity of the local *L. leucocephala* was significantly lower than the *Leucaena* hybrid Bahru, although the molecular weights of CTs of the two were nearly similar. This suggested that, apart from molecular weight, the ability of CTs to interact with proteins to form CT-protein complexes may be associated with other factors, such as the chemical structure of CTs [22,23,24,25,26]. However, the determination of the chemical structure and molecular mass distribution of CTs is challenging due to their complexity and diversity. It has been suggested that the relationship between molecular weight and chemical structure of CTs on protein-binding abilities could be studied by the fractionation of CTs [21,27,28]. Huang *et al.* [26] studied the protein-binding affinity among five CT fractions of different molecular weights of *Leucaena* hybrid Bahru and reported that the larger molecular weight fractions exhibited stronger protein-binding affinity than the smaller molecular weight fractions.

Recently, pure CTs extracted from *L. leucocephala* hybrid Rendang (LLR) were found to decrease enteric ruminal methane emissions, and reduced nitrogen degradability with no adverse effects on dry matter digestibility *in vitro* [17]. The reduction in nitrogen degradability suggested that the CTs of LLR has an ability to bind protein. However, this protein-binding affinity as well as the structure and molecular weight of CTs of LLR have not been studied. In this study, we aimed to fractionate the CTs of LLR into fractions of different molecular weights, determine the distribution of degrees of polymerization (DP) of the CT fractions, characterize the structure of CT fractions by ^13^C-NMR, determine the molecular weights of the fractions by Q-TOF LC-MS, and determine the protein-binding affinities of the CT fractions.

## 2. Results and Discussion

### 2.1. Extraction, Purification and Fractionation of CTs

**Figure 2:**
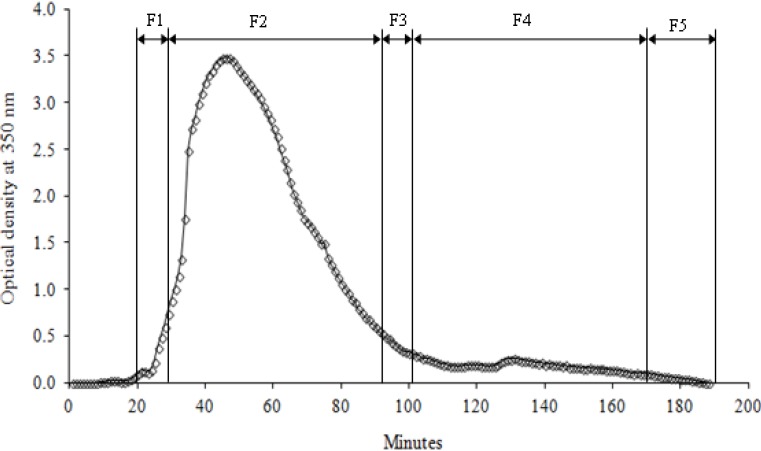
Size exclusion chromatograms of CTs from *L. leucocephala* fractionated using Sephadex G-25. The detector was set to 350 nm and the elution time was over 200 min. Fractions were combined according to their absorbance.

The elution profile of the purified CTs in Sephadex G-25 using 50% acetone as eluent is shown in Figure 2. In this fractionation system, CT extract did not bind to the gel and was fully eluted with 50% acetone, as indicated by the zero reading of the spectrophotometer (350 nm) at the end of the fractionation cycle. This elution profile was reproducible for the pure CTs extracted from all the batches of young leaves and shoots of LLR.

There was a 5-fold reduction in yield after crude CT extract from LLR was purified, as 30% DM of crude CTs yielded 6.2% DM of pure CTs. As a result, the amount of pure CTs obtained from crude CTs was 20.7% on a DM basis. The yields of CTs obtained from each fraction using Sephadex G-25 are summarized in Table 1. It is evident that fraction 2 had the highest yield (74.9%) from the total purified CTs, followed by fraction 1 (10.1%), fraction 3 (8.5%), fraction 4 (4.2%), and the lowest yield was from fraction 5 (2.3%).

The present study demonstrated the application of size exclusion chromatography (Sephadex G-25) in the fractionation of purified CTs on the basis of their size, although the molecular weight determination would be required to confirm this. Fractionation of CT polymers by size exclusion chromatography using Sephadex G-25 with 50% acetone as eluent has also been carried out by Huang *et al*. [26].

**Table 1:** Yields of purified and fractionated CTs from LLR by size exclusion chromatography.

Sample	Yield (% DM)	SEM
Purified CTs *^a^*	20.7	1.21
Fraction 1 *^b^*	10.1	0.62
Fraction 2 *^b^*	74.9	2.46
Fraction 3 *^b^*	8.5	0.57
Fraction 4 *^b^*	4.2	0.34
Fraction 5 *^b^*	2.3	0.21

*^a^* Based on crude CTs. *^b^* Based on purified CTs. SEM: Standard error of the mean.

### 2.2. Degrees of Polymerization of CT Fractions

**Figure 3:**
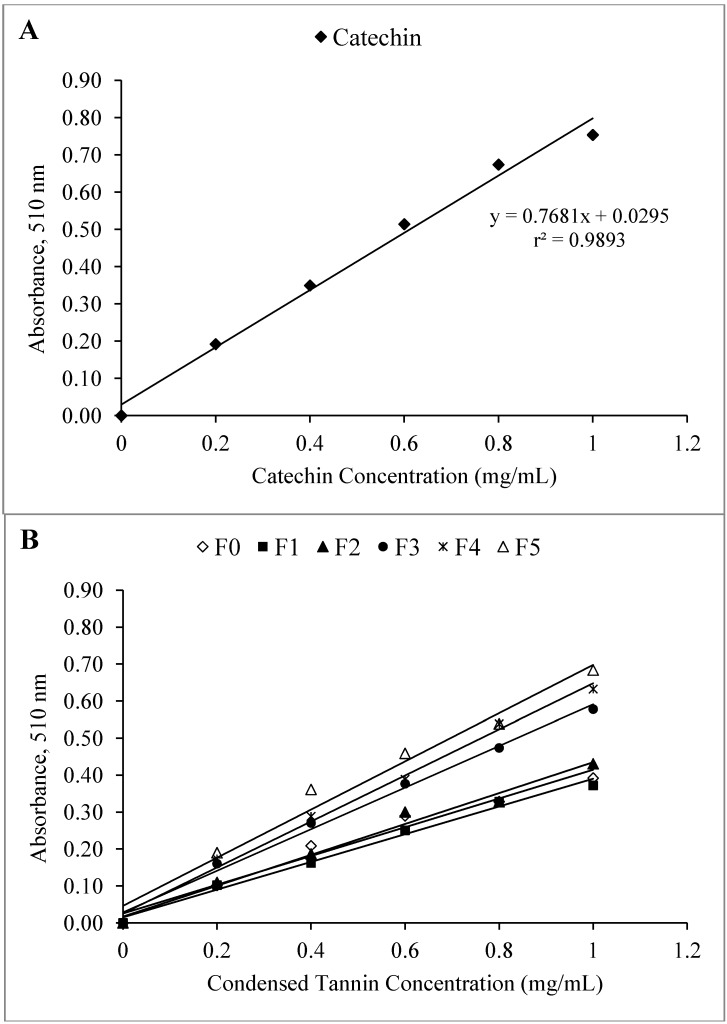
Standard curve (**A**) of the reaction of catechin with vanillin in glacial acetic acid and sample curves (**B**) of the reaction of CT fractions with vanillin in glacial acetic acid. The standard deviations (SD) for catechin (**A**) and samples (**B**) in the vanillin assay were below 0.02.

The DP of the CT fractions were determined from the absorbance measured by the modified vanillin assay in glacial acetic acid for equal weights of CT fractions and tannin monomer (catechin) (Figure 3). Butler *et al.* [29] have reported that, in glacial acetic acid, the reactions of CTs and catechin with vanillin are kinetically similar. These similar kinetics are probably the result of the specificity of the reaction between vanillin and CTs in glacial acetic acid [30]. However, the measurement of the DP as described above requires purified CTs, which can be weighed to determine the total number of flavan-3-ol units. The results for the estimated DP are shown in Table 2. Between 1 and 5 monomer units of catechin per polymer of different fractions of CTs (F1–F5) were obtained, indicating that CTs from LLR possessed structural heterogeneity due to the presence of monomer and oligomers of flavonoid units. The DP of CT fractions decreased gradually from fraction F1 to fraction F5, with values of 4.86, 3.60, 2.50, 2.34, and 1.56 for fractions F1, F2, F3, F4, and F5, respectively. These results suggest that the molecular weight of CTs decreased gradually from fraction F1 to fraction F5. The DP of the fractionated CTs indicated their polymeric nature, and it appeared that their separation on Sephadex G-25 with 50% (v/v) acetone as eluent is primarily dependent on their molecular size. Kumar and Horigome [6] have reported similar absorption spectrum from the vanillin assay for 5 CT fractions of *Robinia pseudoacacia* leaves when subjected to Sephadex LH-20 with 50% (v/v) acetone as eluent; the DP of the 5 fractions obtained ranged from 1.53 to 4.12. Muchuweti *et al.* [31], who also used the modified vanillin assay method, have determined 4 to 10 monomer units of catechin per CT polymer in some wild fruits from Zimbabwe.

**Table 2:** Estimated DP of CT fractions from LLR by the modified vanillin assay and linear regression statistics of the absorption spectrum curves.

CT Fraction	Slope	Intercept	*r*^2^	DP
F1	0.375	0.017	0.99	4.86 *^a^*
F2	0.417	0.014	0.98	3.60 *^b^*
F3	0.562	0.028	0.99	2.50 *^c^*
F4	0.622	0.025	0.99	2.34 *^c^*
F5	0.651	0.029	0.98	1.56 *^d^*

*^a^*^–*d*^ Means within a column with no common superscript differ significantly (*p* < 0.05). *r*^2^: Coefficient of determination.

The vanillin assay reaffirmed that CTs from LLR could be fractionated by size exclusion chromatography. However, since only the terminal units of CTs react with vanillin in glacial acetic acid leading to lower apparent molecular weights, Hagerman [30] has pointed out that this method has not been validated for branched chain CTs. Therefore, although the modified vanillin assay revealed the estimated DP of the CT fractions, the complex structural characteristics and molecular weights of CT fractions cannot be reliably obtained from the assay.

### 2.3. ^13^C-NMRAnalysis of CT Fractions

**Figure 4:**
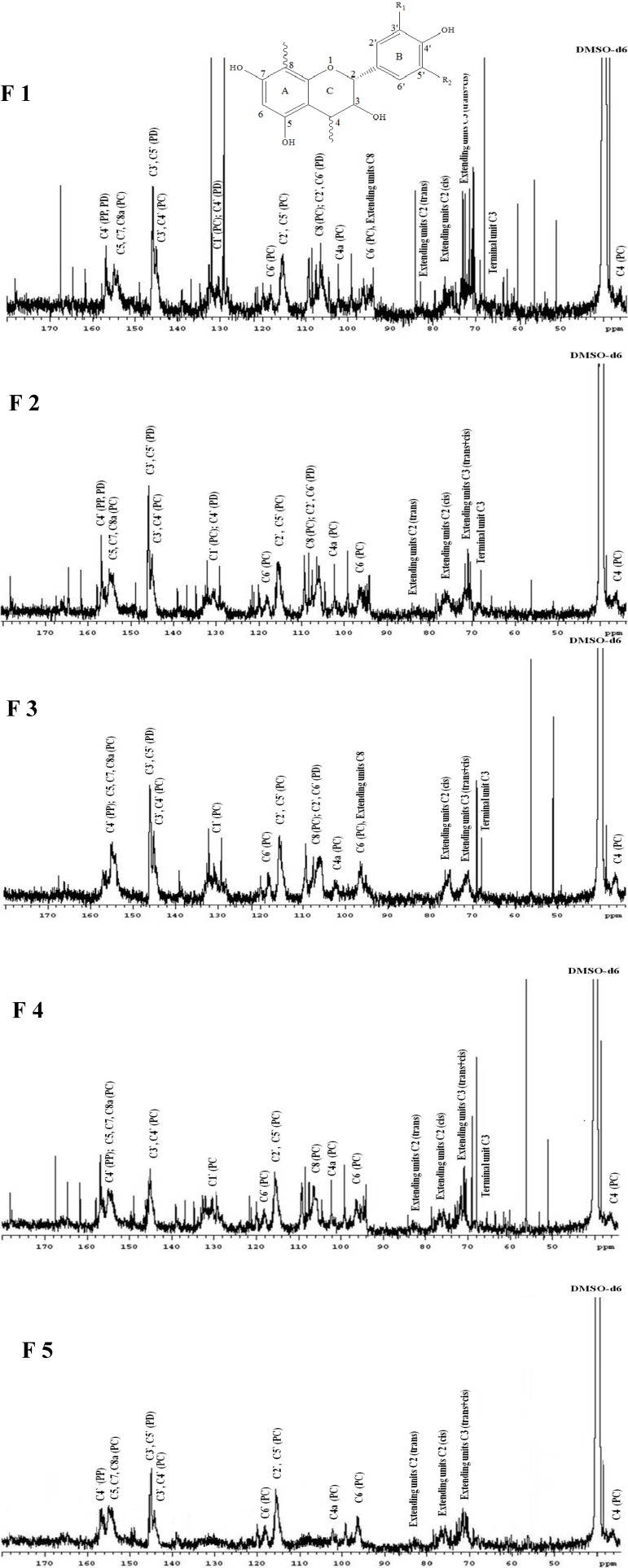
^13^C-NMR (125 MHz) spectra of CT fractions F1, F2, F3, F4 and F5 from LLR in DMSO-*d*_6_; DMSO-*d*_6_, dimethyl-*d*_6_ sulfoxide. Identity of the structures: R_1_=R_2_=H, propelargonidin (PP); R_1_=OH, R_2_=H, procyanidin (PC); and R_1_=R_2_=OH, prodelphinidin (PD).

The ^13^C-NMR spectra of CTs have been employed to present information on structures of the chain terminating flavan-3-ol units, the ratio of procyanidin (PC) to prodelphidin (PD) units, the number-average molar mass (*M*_n_) and the stereochemistry of the CT unit [32]. The ^13^C-NMR spectra of the different molecular weight fractions of CTs (F1–F5) from LLR are shown in Figure 4. The signal assignment was performed according to relevant literature reports [1,32,33,34]. The ^13^C-NMR spectra of the different molecular weight factions of CT (F1, F2, F3, F4 and F5) in DMSO-*d*_6_ show characteristic peaks consistent with those of CTs with PC (catechin/epicatechin), PD (gallocatechn/epigallocatechin) and propelargonidin (PP) (epiafzelechin/afzelechin) units. PC can be distinguished from PD because PD has a hydroxyl group at position 5 of the B ring. The clusters of characteristic peaks between 170 to 30 ppm show that all five CT fractions are composed of predominantly PC units and a minor amount of PD units. Other studies have also shown that the majority of CTs consist of more PC than PD units [1,33,34]. It has been reported that PC and PD units have been considered as functional ingredients in nutritional supplements [35].

The stereoisomers and structural diversity of the linkages (A and B type) of PC, PP and PD units are apparent from the spectra (Figure 4). For all five fractions (F1–F5), peaks at 157 to 150 ppm are assignable to the C4' of PP units, and C5, C7 and C8a carbons of PC units. The spectrum signals at 145.2 and 145.3 ppm belong to C3' and C4' of PC units. The signals at 115.1, 115.5, and 118.2 ppm are assignable to the C2', C5' and C6' of PC units. The C3' and C5' of PD units show typical resonance at 146 ppm for fractions F1, F2, F3 and F5 but are not found in fraction F4. This shows that fractions F1, F2, F3 and F5 are composed of PP, PC and PD units but fraction F4 is composed of only PP and PC units. The PD/PC ratio of CTs is determined from the relative ratio of the peak areas at 145 ppm (C3' and C4' of PC) and 146 ppm (C3' and C5' of PD) [34]. Table 3 shows the PD/PC ratio of the CT fractions. The PD/PC ratio is highest for fraction F1 as compared to the other CT fractions. An increase in the PD/PC ratio in a CT increases the ability of that CT to complex with protein [15]. A small amount of PD is also detected at its C4' peak, which appears at 131 ppm in fractions F1 and F2, overlapping with the chemical shifts of C1 carbon of PC unit. The peak clusters between 110 and 90 ppm in fractions 1 to 3 are assigned to C8, C4a and C6 of PC, and C6' and C2' of PD; for fraction F4 only C8, C4a and C6 of PC appear but C6' and C2' carbons of PD are not found; and for fraction 5 only C4a and C6 of PC are observed. The signals which appear between the 30 and 90 ppm region are assigned to carbon C2, C3 and C4 in flavan-3-ol units. The stereochemistry of the C ring is apparent at the region between 70 and 90 ppm. The two peaks at 76 and 83.3 ppm are recognized to be 2,3-*cis* to 2,3-*trans* isomers, respectively. The ratio of the 2,3-*cis* to 2,3-*trans* isomers can be obtained from direct integration of the signals [1,34]. The spectra reveal that both the stereoisomers (*trans* and *cis*) exist together in all the five fractions. From the peak area at 76 ppm, it is estimated that the *cis* isomer is dominant. The percentage of *cis* form is estimated to be 92.6%, 97.5%, 100%, 80.4% and 68.1% for fractions F1, F2, F3, F4 and F5, respectively. The signal at 71.5 ppm is assignable to the C3 of both *trans* and *cis*. A distinct line at 68 ppm, due to C3 of the terminal unit, occurs in fractions F1, F2, F3 and F4, but this terminal signal is not detected in fraction F5. The terminals to extender ratios are estimated to be 16.3, 15.2, 9.5 and 7.6 for the CT fractions F1, F2, F3 and F4 respectively. At 36 ppm, the C4 carbons of the extension units show a broad peak.

**Table 3:** PD to PC ratios of CT fractions.

Fraction	PD %	PC %	PD/PC ratio
F1	67.3	32.7	2.06
F2	64.8	35.2	1.84
F3	62.0	38.0	1.63
F4	0.0	100	0.00
F5	58.0	42.0	1.38

PD: Prodelphindin; PC: Procyanidin.

### 2.4. Molecular Weight of Different CT Fractions from LLR

Although the ^13^C-NMR spectra revealed the complex structural characteristic of the five CT fractions, quantitative data on their molecular weights and the DP could not be reliably obtained. Further characterization was achieved by Q-TOF LC-MS analysis. Table 4 shows the Q-TOF LC-MS analysis of the five CT fractions from LLR. The mass of ion series of CT fraction F1 to fraction F5 ranged from 444 to 1443 Da. The ion series of CT fractions, recorded as [M+Na]^+^ and [M+H]^+^ adducts in the positive ion reflection mode, was assigned to (epi)catechin, (epi)afzelechin, and (epi)gallocatechin oligomers and galloylated derivatives with mass differences of 288, 272, 304, and 152 Da, respectively. The following equations were applied to predict the mass distribution and the flavan-3-ol unit composition [36]:

[M+Na]^+^ = Na^+^ + 304 *a* + 288 *b* + 272 *c* + 152 *G* − 2.0 *B* − 4.0 *A* + 2.0

[M+H]^+^ = H^+^ + 304 *a* + 288 *b* + 272 *c* + 152 *G* − 2.0 *B* − 4.0 *A* + 2.0

where *a*, *b* and *c* are the number of (epi)gallocatechin, (epi)catechin and (epi)afzelechin units, respectively, *G* is the number of galloylated derivatives attached to the flavan-3-ol units, Na^+^ and H^+^ correspond to the molecular weights of the added cations (*m*/*z* 23 for Na^+^ and *m*/*z* 1 for H^+^), 2.0 is the number of H in the end groups, *B* and *A* are the numbers of B-type and A-type linkages between the units, and *a* + *b* + *c* is the DP deriving from the repeated (epi)gallocatechin, (epi)catechin and (epi)afzelechin units occurring in oligomers and polymers of CTs.

The application of these equations could reveal the presence of a series of CTs consisting of well‑resolved polymers. The results obtained indicated that the masses of the peaks among the CT fraction polymers with identical DP increased at different distance of 288, 272, and 304 Da, corresponding to (epi)catechin, (epi)afzelechin and (epi)gallocatechin oligomers. The galloyl group, which showed the mass signal at 152 Da, was also detected in the mass spectrum, which is similar to the finding reported by Huang *et al.* [26] on another hybrid of *Leucaena*. In the present study, series of peaks with subset mass increments of 16 and 32 Da within the same DP were detected in fractions F1, F2, F3, F4 and F5 (Table 4). These masses can be explained by heteropolymers of repeating flavan-3-ol units containing additional hydroxyl group (Δ 16 Da) at the position 5' of the B-ring [34].

A series of ions corresponding to Na^+^ and H^+^ adducts of CT oligomers and polymers from DP 1 to DP 5 were observed from 444 (Table 4, fraction F5, peak 3) to 1,443 Da (Table 4, fraction F1, peak 4), indicating that CTs of LLR are made up of a complex set of different flavanol units. The structures of the CTs were obtained by determination of the calculated or theoretical mass. The calculated or theoretical mass corresponds to the different classes of CTs, which is calculated as the accumulated number of (epi)catechin, (epi)afzelechin and (epi)gallocatechin oligomers, and galloylated derivatives [36]. Since the absolute masses corresponded to each peak, it indicates that the CT fractions contain PP, PC or PD units, as have already been shown in the ^13^C-NMRspectra (Figure 4). These masses can be explained by homopolymers or heteropolymers of CTs which are composed by the repeating flavan-3-ol units of PP, PC and/or PD. However, this method does not allow us to distinguish the different stereoisomers.

**Table 4:** Composition of the five fractions of CTs identified by Q-TOF LC-MS spectra.

CT		Observed Mass (*m*/*z*)	*I ^a^* (× 10^4^)	Ion Detected	Calculated Mass (Da)	Possible Assignments *^b^*	DP *^c^*
Fraction 1	Peak 1	1223.2250	4.50	[M+Na]^+^	1200	304*3 + 288 + 2	4
	Peak 2	1241.2910	2.00	[M+Na]^+^	1218	304*4 + 2	4
	Peak 3	1291.7166	3.25	[M+H]^+^	1290	288*3 + 272 + 152 + 2	4
	Peak 4	1443.2680	1.00	[M+ H]^+^	1442	288*5 + 2	5
	CT av *M*_n_ *^d^*	1265.8					
Fraction 2	Peak 1	785.5299	0.50	[M+Na]^+^	762	304*2 + 152 + 2	2
	Peak 5	747.5016	0.90	[M+H]^+^	746	(288 + 304 + 152 + 2)	2
	Peak 4	856.5647	0.15	[M+Na]^+^	832	(272*2 + 288 + 2) – 2H	3
	Peak 4	858.5647	0.15	[M+Na]^+^	834	272*2 + 288 + 2	3
	Peak 5	1067.226	0.15	[M+H]^+^	1066	304*3 + 152 + 2	3
	Peak 6	1241.295	2.00	[M+Na]^+^	1218	304*4 + 2	4
	CT av *M*_n_	1028.6					
Fraction 3	Peak 1	611.3065	0.25	[M+H]^+^	610	304*2 + 2	2
	Peak 2	601.1330	0.45	[M+Na]^+^	578	288*2 + 2	2
	Peak 3	633.4391	0.25	[M+Na]^+^	610	304*2 + 2	2
	Peak 4	727.4915	0.27	[M+H]^+^	726	(288*2 + 152 + 2) – 2H	2
	Peak 5	769.5189	0.15	[M+Na]^+^	746	288 + 304 + 152 + 2	3
	CT av *M*_n_	652.2					
Fraction 4	Peak 1	547.3872	0.35	[M+H]^+^	546	272*2 + 2	2
	Peak 2	563.4121	0.30	[M+H]^+^	562	288 + 272 + 2	2
	Peak 3	579.4142	0.32	[M+H]^+^	578	288*2 + 2	2
	CT av *M*_n_	562.2					
Fraction 5	Peak 1	481.2794	0.25	[M+Na]^+^	458	304 + 152 + 2	1
	Peak 2	482.3310	0.60	[M+Na]^+^	459	304 + 152 + 2	1
	Peak 3	444.3079	0.30	[M+H]^+^	443	288 + 152 + 2	1
	Peak 4	461.3260	2.20	[M+Na]^+^	438	(288 + 152 + 2) – 2H	1
	Peak 5	479.3260	2.20	[M+Na]^+^	578	288*2 + 2	2
	Peak 6	611.4354	0.24	[M+H]^+^	610	304*2 + 2	2
	CT av *M*_n_	469.6					

*^a^* *I* = absolute intensity (× 10^4^). *^b^* 304, 288, 272,152 and * represent the calculated molecular weights of (epi)gallocatechin, (epi)catechin/(epi)robinetinidol, (epi)fisetinidol/(epi)afzelechin, and galloyl derivatives, respectively. *^c^* DP, degree of polymerization. *^d^* CTs number-average molecular weights (*M*_n_) was calculated with the equation *M*_n_ = (Σ(*m*/*z*)*_i_I_i_*)/(Σ*I_i_*).

The fractionation of CTs according to their molecular weight improves mass spectral analysis and facilitates the interpretation of the true molecular weight distribution and proportion of the CTs. In the present study, CT fractions of different molecular weights from LLR possessed structural heterogeneity in monomer units and DP. As shown in Table 4, four peaks with molecular weights ranging from 1221 to 1443 Da were detected in the first fraction (F1) with a number-average molecular weight (*M*_n_) of 1265.8 Da, and DP from 4 to 5; the highest molecular weight was detected in peak 4, which might be assigned to isomers of the PC pentamer with 5 units of (epi)catechin. Other peaks of fraction F1 could be assigned to CT polymers consisting of PD or a combination of PC and PD or PP, which was assigned to CT tetramers. The second fraction (F2) consisted of 6 peaks with molecular weights ranging from 741 to 1,241 Da with a *M*_n_ of 1028.6 Da and DP from 2 to 4, which might be assigned to CT polymers consisting of PD or a combination of PC and PD or PP monomers. Five peaks were detected in the third fraction (F3) with molecular weights ranging from 611 to 769 Da. The *M*_n_ of this fraction calculated from the intensities of peaks was 652.2 Da and DP varied from 2 to 3. Fraction F3 consisted mainly of PD dimers with 2 units of (epi)gallocatechin and PC dimers with 2 units of (epi)catechin. The fourth fraction (F4) consisted of 3 peaks with molecular weights ranging from 547 to 577 Da. The *M*_n_ of this fraction was 562.2 Da and DP was 2. Fraction F4 consisted mainly of PC and PP dimers or a combination of PC and PP units which was assigned to CT dimer. Since the absolute masses corresponded to each peak in fraction F4, it suggests that this fraction only contains PC and PP units without PD units, as has been shown in the ^13^C-NMRspectrum (Figure 4). Six peaks were detected in the fifth fraction (F5), with molecular weights ranging from 444 to 611 Da, *M*_n_ of 469.6 Da, and DP ranged from 1 to 2, which were interpreted as galloylated CT dimers and monomers of PD and PC.

Although Sephadex LH-20 has been widely used for proanthocyanidin chromatography since 1974 [37], the above results demonstrated that using a Sephadex G-25 column, the molecular weights of the CTs eluted out decreased from the first to the fifth fraction, thus indicating the successful fractionation of the CTs into different molecular weights. The results from the molecular weight determination by Q-TOF LC/MS showed that fraction F1, which was eluted out first, had the highest molecular weight, followed by fractions F2, F3, F4, and lastly F5, which had the lowest molecular weight. The calculated *M*_n_ values were 1265.8, 1028.6, 652.2, 562.2, and 469.6 Da for the first to the fifth fraction, respectively. Hence, the molecular weights of the fractions were: F1 > F2 > F3 > F4 > F5. The results of the current study indicate that LLR contains CTs with different polymer chain lengths, varying from monomers to pentamers. This is in agreement with the study of Saucier *et al.* [38], who reported the successful separation of CTs with DP up to pentamers using different size-exclusion chromatography gels such as Sephadex G25, LH20, and TSK HW40. However, using the same fractionation method of the present study, Huang *et al.* [26] found DP up to hexamer in CT fractions from another *Leucaena* hybrid. This occurrence indicated that CT composition in plants might be genetically determined because they differ within different plant species.

### 2.5. Protein-Binding Affinity of CTs of Different Molecular Weight Fractions

**Figure 5:**
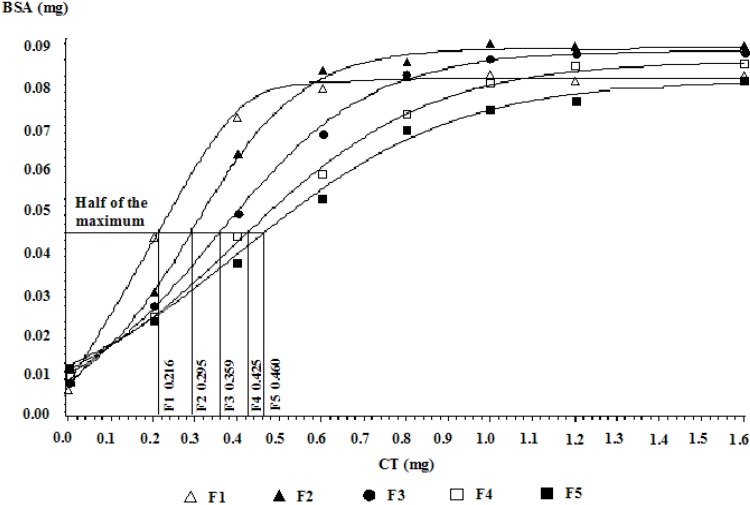
Protein-binding affinities of CT fractions of different molecular weights from LLR. The Y-axis represents the bonded bovine serum albumin (BSA) values, and the X‑axis represents different CT amounts. The standard deviations (SD) for all the CT fractions in the protein binding assay were below 0.002.

Since the physiological activity of CTs is believed to be related to their capacity to bind proteins, the interaction between CTs and proteins has been studied extensively [6,16,20,21]. In the present study, the biological activity of CT fractions of different molecular weights from LLR was evaluated through the analysis of their protein-binding affinity using the protein precipitation assay. In this study, the *b* value, which was the amount of CT used to bind half of the maximum perceptible bovine serum albumin (BSA), was used to indicate the protein-binding affinity of CTs (*i.e.*, the protein-binding affinity of CTs is stronger when the *b* value is smaller). The *b* values of the different molecular weight fractions of CTs are shown in Figure 5 and Table 5. The results showed that the *b* value increased from fractions F1 to F5, with values of 0.216, 0.295, 0.359, 0.425, and 0.460 for F1, F2, F3, F4, and F5, respectively (Figure 5 and Table 5). The *b* value of the first fraction (F1 = 0.216) was significantly lower (*p* < 0.05) than the other fractions, indicating that the protein-binding affinity of the first fraction was the highest (Table 5). On the other hand, the *b* values of the fourth and fifth fractions (F4 = 0.425; F5 = 0.460) were significantly (*p* < 0.05) higher than those of the other three fractions, indicating that their protein-binding affinities were lower. These results suggest that the protein-binding abilities of heterogeneous CTs extracted from LLR are related to the molecular size (Table 5); the higher the molecular weight of CT fractions, the greater the tendency to precipitate protein. Studies on CTs of several species of *Leucaena*, including *L. trichandra*, *L. pallida* and *L. leucocephala* hybrid Bahru have also shown that larger molecular sized CTs have stronger protein-binding capacity than smaller molecular sized CTs [6,26]. Similarly, Kumar and Horigome [6] reported that the protein-precipitating capacity of the CT fractions of *Robinia pseudoacacia* increased with the increase in the DP. Haslam [39] also discovered that larger-sized tannins have the ability to form multiple bonds with proteins compared to smaller-sized tannins. The CT-protein binding is mainly based on hydrophobic interactions and hydrogen bonding [40,41]. Therefore, CTs with higher molecular weight contain a larger number of hydroxyl group to enable the formation of cross-links with proteins [42]. It has been postulated that the binding between proteins and CTs occurs primarily by hydrogen bond formation between the phenolic hydroxyl groups of the CTs and the carbonyl groups of the protein peptide bonds [29,43,44,45,46].Thus, the nature and the number of consecutive units could explain the potential greater protein-binding ability of high molecular weight CT fractions. In particular, the number of hydroxyl groups within the structure of CTs polymers—which depends upon the DP and increases regularly with the increase in molecular size—has a major effect on the degree of binding [47].

Although molecular weight of CTs is an important factor influencing its protein-binding affinity, other factors such as CT structural differences including the interflavonoid bond cleavage or its stereochemical structure, and the relative number of hydroxyl groups on constituents units may also play a role. This is demonstrated by CT fractions F4 and F5 where the molecular weight of fraction F4 (562.6 Da) was much higher than that of fraction F5 (464.6 Da), but their protein-binding affinities (F4, *b* = 0.425; F5, *b* = 0.460) were not significantly different. This could be due to the similarity in their monomeric composition, especially the number of hydroxyl units between fractions F4 and F5, respectively. Although fraction F4 consists mainly of CTs with (epi)catechin/(epi)robinetinidol and (epi)fisetinidol/(epi)afzelechin, and fraction F5 is mainly made up of (epi)catechin/(epi)robinetinidol, (epi)gallocatechin and galloyl derivatives, the numbers of total hydroxyl units of the two fractions were nearly identical, that is, 8 and 9 or 10 for fractions F4 and F5, respectively. The presence of galloyl derivatives that carry hydroxyl units in most CT monomers of fraction F5 could be the reason for the comparable number of hydroxyl units between fractions F4 and F5, resulting in nonsignificant difference in their protein-binding affinities. Similar observations on *Leucaena* hybrid Bahru have been reported by Huang *et al.* [26]. Aerts *et al.* [48] also found that, although the average sizes of CTs from *Lotus pedunculatus* and *L*. *corniculatus* were nearly similar, CTs of *L. pedunculatus* were better able to bind protein than CTs of *L. corniculatus*, possibly owing to differences in structure. As pointed out by Aerts *et al.* [15] different structures as well as different molecular weights of CTs may result in discrepancies in their ability to bind proteins.

**Table 5:** Protein-binding affinities of CT fractions of different molecular weights using bovine serum albumin (BSA) as reference protein.

Fraction	Molecular weight (Da)	*b* value ^1^
F1	1265.80	0.216 ± 0.025 *^a^*
F2	1028.60	0.295 ± 0.036 *^b^*
F3	652.20	0.359 ± 0.049 *^c^*
F4	562.20	0.425 ± 0.022 *^d^*
F5	469.60	0.460 ± 0.048 *^d^*

*^a^*^–*d*^ Means within a column with no common superscript differ significantly (*p* < 0.05). ^1^ *b* value is the CT quantity (mg) that is needed to bind half of the maximum precipitable BSA. It is used to denote the protein-binding affinity of CTs in this study. When *b* value is smaller, the protein-binding affinity of the CTs is stronger.

## 3. Experimental Section

### 3.1. Chemicals and Plant Materials

All chemicals used were of analytical reagent (AR) purity grade. The solvents, *i.e*., acetone, methanol, diethyl ether, acetonitrile and glacial acetic acid, were from Merck (Darmstadt, Germany). Ascorbic acid, formic acid, ninhydrin, sodium acetate, cellosolve, hydrochloric acid, sodium chloride, BSA and DMSO-*d*_6_ were from Sigma-Aldrich (St Louis, MO, USA). Vanillin and catechin were from Sigma‑Aldrich (Steinheim, Germany).

Young leaves and shoots of LLR were harvested from the research farm of the Department of Animal Science, Universiti Putra Malaysia (3°00' 18.88" N, 101°42' 15.05" E) between 09:00 and 10:30 h. The harvested samples were immediately freeze-dried, ground through a 1.0 mm pore size sieve, and stored at −20°C in airtight dark containers prior to extraction.

### 3.2. Extraction and Purification of CTs

CTs were extracted from freeze-dried LLR using acetone and diethyl ether according to Terrill *et al.* [49]. The CTs were extracted from 200 mg of freeze-dried samples with 200 mL of 70% (v/v) aqueous acetone containing 0.1% (w/v) ascorbic acid in a shaker at room temperature for 20 min. The resulting slurries were centrifuged at 3500 *×g* for 10 min to obtain the supernatant. The residues were re‑extracted twice, the supernatants combined and filtered through Whatman no. 1 filter paper in a Buchner funnel under vacuum. The filtrate was partitioned between an equal volume of diethyl ether in a separation funnel in order to remove pigments, chlorophyll, lipophilic compounds and low molecular weight phenolic acids. Traces of acetone and diethyl ether in the extracts were evaporated under vacuum in a Büchi rotary evaporator (Büchi Labortechnic, Flawil, Switzerland) at 40 °C. The extracts were kept in a 500-mL bottle, and an equal volume of 40% (v/v) aqueous methanol was then added. The mixed solution was purified using Sephadex LH-20 (GE Healthcare Bio-Sciences AB, Uppsala, Sweden) packed in a 40 cm × 16 mm i.d. XK16 column (GE Healthcare Bio-Sciences AB), which was first eluted with 40% (v/v) aqueous methanol and then with 80% (v/v) aqueous acetone. Low molecular weight fractions and other polyphenols were eluted with 40% (v/v) aqueous methanol, while the CTs were eluted with 80% (v/v) aqueous acetone. Traces of acetone in the purified CTs were evaporated using a Büchi rotary evaporator at 40 °C, and then CTs were lyophilized and stored at −20 °C in the dark.

### 3.3. Fractionation of Purified CTs

The purified CTs powder was dissolved in 50% (v/v) aqueous acetone to obtain a mixture concentration of 1 mg/mL. The different fractions of CTs were separated using a 40 cm × 16 mm i.d. XK16 column (GE Healthcare Bio-Sciences AB) packed with Sephadex G-25 (GE Healthcare Bio‑Sciences AB). The mixture was eluted from the column in 5 min; about 6 mg of purified CT powder was applied to the column at a flow rate of 1.2 mL/min, which was controlled using a peristaltic pump (Gilson, Villiers le-Bel, France). The column was eluted with 50% (v/v) aqueous acetone as mobile phase for 16 min prior to collection of the analytes (CT fractions). Then, each fraction was collected every 30 s and the absorbance at 350 nm was recorded. All the collected fractions were later grouped into five fractions on the basis of their spectral readings as shown in Figure 2. The five CT fractions with traces of aqueous acetone were evaporated using a Büchi rotary evaporator at 40 °C, and then lyophilized and stored at −20 °C prior to further analysis.

### 3.4. Degree of Polymerization Determination by Modified Vanillin Assay

The DP of the fractionated CTs were determined by the modified vanillin assay in glacial acetic acid as described by Hagerman [30]. Aliquots from 0 to 1.0 mL of catechin (standard) (0.05 mg/mL in glacial acetic acid) and CT fractions (0.1 mg/mL in glacial acetic acid) were dispensed into separate culture tubes. Each sample was brought to 1.0 mL by the addition of glacial acetic acid. The tubes were incubated in a water bath at 30 °C for 5 min to bring them to equilibrium temperature, after which 5.0 mL of freshly prepared vanillin reagent (50:50 mixture of 1% vanillin and 8% HCl in glacial acetic acid) was added at 1.0 min intervals to the samples. The samples were incubated at 30 °C in the water bath for 20 min, and the absorbance was determined at 510 nm. Each sample was analysed in triplicate and the vanillin assay was performed thrice. The absorbance of the blank (reagent with no tannin) was subtracted from the absorbance of the corresponding vanillin-containing sample. Two response curves were constructed, and each was checked for linearity and zero intercept. The absolute DP of the different CT fractions obtained from the modified vanillin assay were calculated using the following equation:

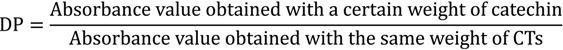


### 3.5. ^13^C-NMRAnalysis

The ^13^C-NMRspectra of different molecular weight fractions of CTs were recorded on a Varian VNMRS-500 (Agilent, Santa Clara, CA, USA) spectrometer at 125 MHz. The samples for recording NMR spectra were prepared by dissolving the samples in DMSO-*d*_6_.

### 3.6. Molecular Weight Determination by Q-TOF LC/MS

Q-TOF LC-MS (Agilent Technologies, Inc.) analysis was performed to determine the number-average molecular weights (*M*_n_) of different molecular weight fractions of the purified CTs. Prior to this analysis, the CT solutions were purified by HPLC. HPLC separation was achieved using a 5-μm Symmetry C18 column, 3.9 × 150 μm (Waters, Wexford, Ireland). Elution was performed using mobile phase A (water containing 0.1% formic acid) and mobile phase B (acetonitrile). The column temperature was maintained at 40 °C and the flow rate was set at 0.5 mL/min. The gradient conditions were 0–20% B, 0–20 min; 20%–40% B, 21–30 min; 40%–100% B, 31–40 min; followed by the return to the initial condition for 20 min. One mL of the purified CT solutions (1 mg/mL) was injected into the Q-TOF LC-MS. The mass spectrometer of the Q-TOF LC‑MS was operated under positive ion mode under the following conditions: dry gas (nitrogen) and sheath gas (nitrogen), at 8 and 11 mL/min, respectively; electrospray ionizer voltage, 3500 V; orifice potential voltage, 40 V. The mass spectra were recorded from *m*/*z* 100 to 3000 with a scanning rate of 1.4. Mass spectral data were processed using Agilent Mass Hunter Workstation Software-Offline Qualitative and Quantitative Analysis. The *M*_n_ of the different CT fractions obtained from Q-TOF LC-MS was calculated using the following equation [26]:

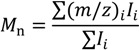

where *I* represents the absolute intensity and *m*/*z* is the mass to charge ratio.

### 3.7. Protein Precipitation Assay

The protein-precipitating ability of CT fractions of differing molecular weights were determined using the procedure of Makkar *et al.* [50] with minor modifications. BSA was used to determine the ability of the CTs to bind proteins. Different concentrations of BSA (0 to 1.2 mg/mL) were used to generate the standard curve. Five fractions of CTs (0.5 mL) at concentrations of 0, 0.2, 0.4, 0.6, 0.8, 1.0, 1.2, and 1.6 mg/mL (prepared in 50% aqueous methanol) were mixed with 0.5 mL BSA buffer (1 mg/mL of BSA in 0.2 M acetate buffer with 0.17 M NaCl at pH 5.0). Each mixture was vortexed and left at room temperature for 15 min to allow the CTs to complex with the BSA and reach the equilibrium. The tubes were then centrifuged at 5000 *×g* for 20 min at room temperature to allow the CT-protein complex to pellet to the wall of the tube. The supernatant was then discarded and the unbound protein was removed by washing with 1.5 mL of 0.2 M acetate buffer. All the tubes were then oven-dried at 100 °C overnight. The hydrolysis was carried out using 13.5 M NaOH at 120 °C for 20 min. After cooling, 100 µL of glacial acetic acid was added slowly and the tubes were vortexed, followed by another addition of 400 and 500 µL of glacial acetic acid and further vortexed to neutralize the solution. Then, 100-µL of this mixture was added to tubes containing 1 mL of ninhydrin solution (0.8 g of ninhydrin, 0.12 g of hydrahydrin, 30 mL of cellosolve and 10 mL of 4 M sodium acetate, pH 5.5). After incubation for 20 min at 100 °C in a water bath, the tubes were cooled, 5 mL of deionized water was added, and absorbance recorded at 570 nm. The protein precipitation assay was carried out with three replicates per sample, and the assay was run three times. Protein binding data were analyzed using a non-linear regression procedure. A graph of precipitated protein *versus* increasing amounts of CTs was produced. The curve relative to CT fractions was fitted with a sigmoid curve: Y = a/(1 + b × exp^(−c × x)^), where Y = mg of BSA precipitated, X = mg of extracted CT fraction incubated. Protein-binding affinity of CTs was expressed as the *b* value that represents the amount of CTs (mg) used to bind half of the maximum BSA.

### 3.8. Statistical Analysis

Statistical analysis of the data was performed by analysis of variance (ANOVA), with significant differences determined by Duncan’s new multiple range test. Statistical significance was considered at *p* < 0.05. All statistical analyses were performed using SPSS for Windows Version 16.0 [51].

## 4. Conclusions

In conclusion, the results of the present study showed that the CTs of LLR could be fractionated into five fractions (F1–F5) of different molecular weights using a size exclusion chromatography procedure. The DP of the CT fractions, determined using a modified vanillin assay in glacial acetic acid, varied from 1 to 5, indicating structural heterogeneity, which was reaffirmed by structural characterization of the CT fractions by ^13^C-NMR, and the calculated molecular weights (*M*_n_) determination by Q-TOF LC-MS. All five CT fractions exhibited protein-binding affinities, with fraction F1, which has the highest molecular weight, exhibiting the strongest protein-binding affinity. As CTs have been suggested to have potential in promoting protein utilization in ruminants [52], higher molecular weight fractions of CTs such as fractions F1 and F2 of LLR should be further studied to exploit their protein-binding affinities to enhance bypass proteins for better utilization of dietary protein in ruminant animals.
